# Supplementary material for: A prediction model for COVID-19 liver dysfunction in patients with normal hepatic biochemical parameters
Source: Life Sci Alliance. 2022 Oct 19;6(1):e202201576. doi: 10.26508/lsa.202201576 (PMC9585965; doi:10.26508/lsa.202201576)
Supplement: Supplementary file 1 [file LSA-2022-01576_TableS1.docx]

# Table S1. Baseline characteristics of the COVID-19 patients in the discovery cohort.

|  |  | **Liver dysfunction** | |  |
| --- | --- | --- | --- | --- |
| **Characteristic** | **Total** | **No** | **Yes** | **P-value** |
| **No.** | 98 | 46 (46.9%) | 52 (53.1%) |  |
| **Sex** |  |  |  | 0.004 |
| Male | 47 (48.0%) | 15 (32.6%) | 32 (61.5%) |  |
| Female | 51 (52.0%) | 31 (67.4%) | 20 (38.5%) |  |
| Age (years) | 38.5 (29.0-51.0) | 37.0 (25.2-51.0) | 40.5 (29.0-51.5) | 0.607 |
| BMI (Kg/m^2^) | 22.2 (20.3-24.8) | 21.1 (19.5-23.8) | 23.0 (20.6-25.4) | 0.029 |
| Smoking | 7 (7.1%) | 4 (8.7%) | 3 (5.8%) | 0.575 |
| Highest temperature | 37.6 (37.0-38.0) | 37.6 (36.9-38.0) | 37.7 (37.2-38.1) | 0.334 |
| SBP | 127.0 (116.0-136.0) | 122.5 (115.2-135.0) | 130.0 (116.0-136.2) | 0.725 |
| DBP | 76.0 (69.0-85.8) | 76.0 (69.0-87.0) | 76.5 (70.0-85.0) | 0.858 |
| **Drugs** |  |  |  |  |
| Antivirus | 17 (17.3%) | 8 (17.4%) | 9 (17.3%) | 0.991 |
| Antibiotic | 18 (18.4%) | 9 (19.6%) | 9 (17.3%) | 0.773 |
| **Comorbidities** |  |  |  |  |
| HBV | 6 (6.1%) | 2 (4.3%) | 4 (7.7%) | 0.491 |
| Hypertension | 11 (11.2%) | 5 (10.9%) | 6 (11.5%) | 0.917 |
| CVD | 2 (2.0%) | 2 (4.3%) | 0 (0.0%) | 0.129 |
| Tumor | 2 (2.0%) | 0 (0.0%) | 2 (3.8%) | 0.179 |
| HIV | 0 (0.0%) | 0 (0.0%) | 0 (0.0%) |  |
| HCV | 0 (0.0%) | 0 (0.0%) | 0 (0.0%) |  |
| COPD | 0 (0.0%) | 0 (0.0%) | 0 (0.0%) |  |
| Diabetes | 4 (4.1%) | 2 (4.3%) | 2 (3.8%) | 0.900 |
| **Symptoms** |  |  |  |  |
| Cough | 55 (56.1%) | 24 (52.2%) | 31 (59.6%) | 0.459 |
| Rhinorrhea | 5 (5.1%) | 3 (6.5%) | 2 (3.8%) | 0.548 |
| Fever | 64 (65.3%) | 31 (67.4%) | 33 (63.5%) | 0.683 |
| Diarrhea | 7 (7.1%) | 3 (6.5%) | 4 (7.7%) | 0.822 |
| Rigor | 1 (1.0%) | 0 (0.0%) | 1 (1.9%) | 0.350 |
| Nausea | 0 (0.0%) | 0 (0.0%) | 0 (0.0%) |  |
| Dyspnea | 3 (3.1%) | 2 (4.3%) | 1 (1.9%) | 0.487 |
| Muscular soreness | 7 (7.1%) | 1 (2.2%) | 6 (11.5%) | 0.072 |
| Feebleness | 14 (14.3%) | 3 (6.5%) | 11 (21.2%) | 0.039 |
| Headache | 10 (10.2%) | 6 (13.0%) | 4 (7.7%) | 0.382 |
| Chest congestion | 8 (8.2%) | 5 (10.9%) | 3 (5.8%) | 0.357 |
| Sore throat | 23 (23.5%) | 11 (23.9%) | 12 (23.1%) | 0.922 |
| Sputum | 24 (24.5%) | 11 (23.9%) | 13 (25.0%) | 0.901 |
| **Chest CT** |  |  |  | 0.461 |
| Single pneumonia | 35 (35.7%) | 19 (41.3%) | 16 (30.8%) |  |
| Double pneumonia | 49 (50.0%) | 22 (47.8%) | 27 (51.9%) |  |
| **Clinical classification** |  |  |  | 0.410 |
| Asymptomatic | 1 (1.0%) | 0 (0.0%) | 1 (1.9%) |  |
| Mild | 18 (18.4%) | 10 (21.7%) | 8 (15.4%) |  |
| Moderate | 74 (75.5%) | 35 (76.1%) | 39 (75.0%) |  |
| Severe | 5 (5.1%) | 1 (2.2%) | 4 (7.7%) |  |
| **Measurements^*^** |  |  |  |  |
| Total | 397 | 159 (40.0%) | 238 (60.0%) |  |
| Per patient | 3.0 (2.0-5.0) | 2.0 (1.0-4.0) | 3.0 (2.0-5.0) | 0.022 |
| **Follow-up time** | 17.0 (10.0-31.0) | 16.0 (10.0-31.0) | 19.0 (10.2-31.0) | 0.516 |

Data are n (%) or median (IQR) unless otherwise indicated.

The asterisk (^*^) represents the test times of hepatic biochemical parameters in total.

Abbreviations: BMI, body mass index; SBP, systolic blood pressure; DBP, diastolic blood pressure; CVD, cardiovascular disease; COPD, chronic obstructive pulmonary disease.
