# Supplementary material for: A prediction model for COVID-19 liver dysfunction in patients with normal hepatic biochemical parameters
Source: Life Sci Alliance. 2022 Oct 19;6(1):e202201576. doi: 10.26508/lsa.202201576 (PMC9585965; doi:10.26508/lsa.202201576)
Supplement: Supplementary file 2 [file LSA-2022-01576_TableS2.docx]

# Table S2. Characteristics of the COVID-19 patients in the external cohort.

|  |  | **Liver dysfunction** | |  |
| --- | --- | --- | --- | --- |
| **Characteristic** | **Total** | **No** | **Yes** | **P-value** |
| **No.** | 13 | 6 (46.2%) | 7 (53.8%) |  |
| **Sex** |  |  |  | 0.002 |
| Male | 6 (46.2%) | 1 (16.7%) | 5 (71.4%) |  |
| Female | 7 (53.8%) | 5 (83.3%) | 2 (28.6%) |  |
| Follow-up time | 22.0 (15.2-32.8) | 23.5 (16.8-32.8) | 22.0 (14.8-31.8) | 0.904 |
| Measurements^*^ | 2.0 (1.0-3.0) | 2.0 (1.0-3.0) | 2.0 (1.0-3.0) | 0.568 |

Data are n (%) or median (IQR) unless otherwise indicated.

The asterisk (^*^) represents the test times of hepatic biochemical parameters in total.
