# Supplementary material for: A prediction model for COVID-19 liver dysfunction in patients with normal hepatic biochemical parameters
Source: Life Sci Alliance. 2022 Oct 19;6(1):e202201576. doi: 10.26508/lsa.202201576 (PMC9585965; doi:10.26508/lsa.202201576)
Supplement: Supplementary file 3 [file LSA-2022-01576_TableS3.docx]

# Table S3. Diagnostic criteria of liver dysfunction.

| **Normal liver function** | AST <35 U/L combined with ALT <40 U/L, GGT <45 U/L, and TBIL <20.52 µmol/L |
| --- | --- |
| **Mild liver dysfunction** | AST ≥35 U/L combined with ALT <40 U/L, GGT <45 U/L, and TBIL <20.52 µmol/L |
| **Moderate liver dysfunction** | AST ≥35 U/L combined with ALT: 40-120 U/L, GGT: 45-90 U/L, or TBIL: 20.52-41.04 µmol/L |
| **Severe liver dysfunction** | AST ≥35 U/L combined with ALT ≥120 U/L, GGT ≥90 U/L, or TBIL ≥41.04 µmol/L |
| **LD** | Any grade of liver dysfunction |

Abbreviations: LD, liver dysfunction; ALT, alanine aminotransferase; AST, aspartate aminotransferase; GGT, γ-glutamyl transferase; TBIL, total bilirubin.
